# Enhancing wellness: a systematic review of biofeedback interventions for healthcare professionals

**DOI:** 10.3389/fpsyt.2026.1761371

**Published:** 2026-03-16

**Authors:** Elisa Cantone, Antonio Urban, Alessandra Perra, Giulia Cossu, Massimo Tusconi, Serdar M Dursun, Mauro Giovanni Carta

**Affiliations:** 1 PhD Course Capacities Building for Global Health, University of Cagliari, Cagliari, Italy; 2 Department of Medical Sciences and Public Health, University of Cagliari, Cagliari, Italy; 3 University Hospital of Cagliari, Cagliari, Italy; 4 Neurochemical Research Unit, Department of Psychiatry University of Alberta, Edmonton, AB, Canada

**Keywords:** biofeedback, healthcare professionals, self-regulation, well-being, work-related stress

## Abstract

**Background/objectives:**

Healthcare professionals are routinely exposed to high psychosocial and physiological demands, placing them at elevated risk for stress-related disorders, including burnout, anxiety, and impaired autonomic regulation. Biofeedback has emerged as a promising non-pharmacological approach to enhance self-regulation and resilience. This systematic review aimed to evaluate the effectiveness of biofeedback-based interventions in improving psychological and physiological outcomes among healthcare workers.

**Methods:**

A systematic search was conducted across PubMed, Cochrane, Embase, PsycINFO, and grey literature (December 2023–January 2024), following PRISMA guidelines and a registered PROSPERO protocol (CRD42024544687). Eligible studies included randomized controlled trials, quasi-experimental designs, and pre-post studies involving adult healthcare workers exposed to work-related stress. Primary outcomes comprised stress, burnout, anxiety, depression, resilience, and physiological indices such as heart rate variability (HRV), respiratory rate, and skin conductance. Data were synthesized narratively due to methodological heterogeneity. Twelve studies met inclusion criteria. HRV-biofeedback and respiratory sinus arrhythmia training demonstrated.

**Results:**

consistent improvements in perceived stress, anxiety, depressive symptoms, emotional regulation, and resilience. Physiological benefits included increased HRV, decreased sympathetic arousal, and improved autonomic balance. Interventions integrating breathing or mindfulness techniques exhibited the strongest effects. However, non-randomized designs and small samples limited the robustness of findings. Discussion/Conclusions: Biofeedback represents a feasible and potentially effective strategy for mitigating occupational stress and enhancing psychophysiological well-being in healthcare professionals. Despite promising results, evidence remains preliminary due to heterogeneity, limited methodological rigor, and scarce long-term follow-up. Future large-scale randomized trials with standardized protocols are needed to strengthen the evidence base and support implementation in occupational health settings.

**Systematic Review Registration:**

https://www.crd.york.ac.uk/prospero/, identifier CRD42024544687.

## Introduction

Healthcare Workers (HCWs) frequently face substantial stressors, including extended working hours, low staff turnover, persistent personnel shortages, and the emotional burden of patient care. Like any large organization, the healthcare system is influenced by several factors that the European Agency for Safety and Health at Work (EU-OSHA, 2014) (1, 2) calls “risk factors”: conflicting demands and unclear roles; limited involvement in decision-making processes affecting workers; little control over task execution; poorly managed organizational changes; job insecurity; ineffective communication; inadequate support from managers or colleagues; psychological and sexual harassment; and, finally, demanding clients, patients, or students (1).

In the literature, we find that several systematic reviews have already shown that healthcare professionals experience high levels of psychological distress, including burnout, anxiety, and depression (3, 4). Several other studies also highlight that dissatisfaction with limited organizational resources and insufficient institutional support significantly contributes to stress and reduces well-being among professionals (5, 6). During and after the COVID-19 pandemic, these conditions worsened, with a notable increase in reports of burnout, distress, anxiety, and depression, mainly due to resource shortages, work overload, and organizational pressures (7, 8). Overall, the evidence suggests that although stress and burnout were common in healthcare before COVID-19, the pandemic greatly intensified these issues, worsening the psychological burden associated with resource limitations and high emotional demands (9, 10).

Currently, the structure of healthcare delivery is becoming more complex, exposing HCWs to more extended periods of intense stress and increasing the risk of adopting dysfunctional and maladaptive coping strategies (11). Under these conditions of high workload, the threshold for well-being is lowered, and symptoms such as anxiety, loss of motivation, mental and physical fatigue, and both personal and professional dissatisfaction may appear, often accompanied by feelings of helplessness and frustration (12, 13). High levels of frustration, particularly, play a key role in the development of burnout, especially among workers who receive limited support from their organization (13).

When these resources are seen as insufficient, HCWs become more susceptible to psychological and physical distress. This distress raises the chances of interpersonal difficulties with colleagues, undermines the ability to maintain assertive and collaborative behavior, and compromises the quality of professional interactions (13, 14). Workers in this situation often feel mistreated and unsupported by their organization, with leadership also viewed as lacking (12, 15). As a result, the organizational climate is experienced as hostile, marked by high emotional stress and professional demands that surpass available personal resources (15).

When the relationship between hospital staff and patients becomes ineffective, patients may lose trust, which in turn can reduce adherence to recommended treatments (16). For healthcare organizations, this may result in increased absenteeism or, conversely, presenteeism, where employees attend work despite being ill and unable to perform effectively (17). Even in structurally well-organized healthcare environments, conditions of distress may emerge if insufficient attention is given to emotional well-being (18). This condition compromises both physical and mental health, with severe consequences for the quality of life (QoL) of HCWs and their patients. Beyond mental health issues such as burnout, anxiety, depression, and even suicidal ideation, prolonged occupational stress also increases the risk of severe physical health conditions, including cardiovascular diseases and stroke (19). Chronic stress hyperactivates the sympathetic nervous system, triggering primitive physiological responses designed to ensure survival. Heart rate and blood pressure rise, and the body prepares to respond through fight-or-flight or freeze responses (20–22). At the same time, parasympathetic activity is inhibited, leading to progressive physiological wear and tear. According to polyvagal theory, sustained sympathetic dominance and vagal withdrawal impair emotional regulation, social engagement, and physical health (20, 23).

Implementing the European Framework Agreement of October 8, 2004, on work-related stress (14) requires employers to assess and manage stress in the workplace. Employers are legally responsible for ensuring that occupational risks are correctly evaluated and effectively mitigated. This regulatory framework underscores the importance of adopting a structured and preventive approach to addressing psychological and physical distress in professional settings. In line with this, the EU Strategic Framework on Health and Safety at Work 2021–2027 (24) further emphasizes the need to address psychosocial risks and promote mental well-being at work, underscoring the importance of preventive, evidence-based approaches to occupational stress management. Recent literature has identified several strategies as effective for managing occupational stress in such settings (25, 26).

Among these, Biofeedback has emerged as a promising physiological self-regulation technique capable of modulating heart rate variability (HRV) and supporting balance of the autonomic nervous system (14, 27). This approach is grounded in the contemporary psychophysiological framework of self-regulation, which integrates neurovisceral and biopsychosocial models of stress and adaptation (14, 23, 26, 28). Biofeedback enhances physiological regulation, bodily awareness, resilience, and cognitive performance, especially with breathing or mindfulness techniques (23, 29). It is a user-friendly, non-invasive, non-pharmacological intervention used for neuropsychological conditions like anxiety, stress, and emotional dysregulation. With no reported side effects, it helps individuals actively maintain health and develop adaptive coping strategies, improving energy and cognitive performance (14, 26, 28, 30). By providing real-time monitoring and feedback of key biomedical signals, such as electromyographic activity (EMG), skin temperature (ST), electrodermal activity (EDA), respiratory rate (RR), heart rate (HR), heart rate variability (HRV), blood pressure (BP), electroencephalographic activity (EEG), and peripheral blood flow (PBF), biofeedback provides real-time sensory feedback (visual, auditory, tactile), increasing awareness and voluntary control of autonomic functions, thus enhancing emotional regulation and physiological balance (31, 32). Various forms of biofeedback, often combined with rehabilitative or behavioral approaches, target medical and psychological conditions (33). Key types of particular relevance to this review include Heart Rate Variability Biofeedback (HRV-BF), Respiratory Sinus Arrhythmia Biofeedback (RSA-BF), Electromyographic Biofeedback (EMG-BF), Neurofeedback, an electroencephalographic biofeedback (EEG-BF) technique, Cardiovascular Biofeedback (CBKF), and wearable postural feedback systems. Despite differences, they all aim to promote psychophysiological balance through feedback learning (26). Biofeedback is effective in reducing chronic fatigue, anxiety, depression, and pain (34).

Beyond its use in clinical settings, biofeedback has been explored with non-clinical groups, including students, athletes, and workers. It has been found to support psychological well-being, resilience, and cognitive efficiency (14, 28, 35). These results emphasize that biofeedback not only helps reduce stress-related symptoms but also encourages optimal functioning, aligning with the idea of psychophysiological flourishing. By improving self-regulation and interoceptive awareness, biofeedback aids in maintaining focus, emotional stability, and energy balance, key factors in job performance and engagement (28, 36). This proactive and empowering role is especially significant for HCWs, who, although not in clinical settings, face ongoing interpersonal challenges and emotional strain. Enhancing their self-regulation through biofeedback training could boost their well-being and job performance while decreasing the risk of stress and burnout.

However, despite the growing body of evidence supporting its use in the general population, no systematic review to date has specifically examined its application among HCWs (32, 33). This gap in the literature provides the rationale for the present review, which aims to systematically evaluate the effectiveness of biofeedback-based interventions in healthcare settings. Accordingly, the present systematic review aimed to address the following research question, structured according to the PICOS framework: among adult healthcare professionals exposed to work-related stress (Population), do biofeedback-based interventions, including HRV-biofeedback, respiratory, electromyographic, or neurofeedback approaches (Intervention), compared with no intervention or alternative stress-management strategies (Comparison), improve mental health and psychological outcomes, such as stress, burnout, anxiety, depression, and resilience, and physiological indicators of autonomic regulation (Outcomes), across experimental and pre-post study designs (Study design).

The review focuses on their impact on psychological and mental health outcomes and physical well-being, as well as on the quality of life of HCWs. It is anticipated that effective biofeedback use may alleviate work-related stress and burnout symptoms, and that this review will offer an overview of the most promising clinical applications for this population.

## Methods

### Protocol and registration

The systematic review was registered in the International Prospective Register of Systematic Reviews (PROSPERO; https://www.crd.york.ac.uk/prospero) under protocol number CRD42024544687. The study was conducted in accordance with PRISMA guidelines and the PICOS framework (37).

### Eligibility criteria

The eligibility criteria followed the PICOS model (Population, Intervention, Comparison, Outcome, Study design): Adults (≥18 years, any gender) employed in the healthcare sector, including healthcare managers with high-level responsibilities, healthcare assistants, nurses, and physicians. Intervention: Biofeedback training (e.g., HRV-BF, EMG, Neurofeedback). Studies combining biofeedback with other interventions were included if the specific effect of biofeedback could be isolated. Interventions not based on biofeedback were excluded; Comparison: No restrictions applied; Outcomes: Mental Health and Psychological outcomes (stress, burnout, anxiety, depression, psychosomatic symptoms, pain, work well-being, efficiency, job satisfaction, resilience) and physiological outcomes (HRV, heart rate, blood pressure, skin conductance, salivary cortisol); Study design: Pre–post studies, feasibility studies, quasi-experimental studies, controlled studies, and randomized controlled trials. Only published studies with accessible full texts were considered.

Exclusion criteria included: participants who were not healthcare professionals or students, interventions other than biofeedback, duplicate records, irrelevant studies (i.e., not addressing stress-related or psychosomatic symptoms), and studies unavailable despite attempts to contact the corresponding author. No restrictions were applied regarding the time frame or language of publication.

### Information sources

The literature search was conducted between December 2023 and January 2024 across the following databases: PubMed/MEDLINE, Cochrane Library, Embase, and PsycINFO.

Gray literature was searched using ProQuest (38). References of retrieved articles, relevant studies, and systematic reviews were examined to identify additional potentially eligible studies. Five additional studies were identified through other sources, of which two were deemed eligible.

When studies were not available, the corresponding authors were contacted directly. The last search was in January 2024 (**Table 1**).

**Table 1:** Summary of the clinical results of the studies reporting the use of Intervention Biofeedback in the healthcare population.

Autor, country, year	Aim of study	Sample	Health workers	Study design	N group (IG: experimental and CG: control group)	Biofeedback intervention type	Measuring instruments	Main results
Munafò (39); Italy; 2016	Assess the effectiveness of a short Training RSA-BF versus a standard stress management intervention in improving psychological and physiological well-being.	31 managers Males aged 35–73 years (mean age = 48.37 ± 8.71)	Managers and middle managers from the health care Other Sectors: Private: banks, manufacturing industries, media Public: military, schools, local government	RCT	IG = 16 CG= 15	Training RSA-BF Training: five weekly sessions, each lasting about 45 minutes, in which participants synchronized heart rate with abdominal breathing using real-time biofeedback. The participants to breathe at a slow and optimal rate to increase respiratory sinus arrhythmia (RSA) and improve vagal tone and autonomic balance. control group: stress diary	**Mental Health and Psychological**: State and trait anxiety: STAI-Y. health status: SF-36 **Physiological**: Blood volume pulse (HR e HRV (lnRSA)): Photoplethysmographic detection sensor (Flex/Pro) attached to the right ring finger; Respiration: Respiratory belts (Respiration-Flex/Pro sensor) worn around the participant’s thorax and abdomen; Systolic and diastolic Blood Pressure (SBP and DPB): (NAIS EW272, Matsushita Electrics Works Italia S.r.l.) on the left arm; Skin conductance level (SCL): Ag/AgCl surface electrodes (Skin conductance-Flex/Pro sensor) applied on the first and middle finger of the right hand.	RSA-BF training significantly improved autonomic regulation, with increased cardiac variability (lnRSA; p = .04) and decreased SCL (p <.001), indicating reduced sympathetic arousal. In addition, only the RSA-BF group showed a significant reduction in work limitations due to emotional problems, alongside general improvements in perceived health and vitality.
Seiça (40); Portugal; 2023	Evaluate whether the biofeedback intervention of Qigong can be used as a complementary therapy for emotional exhaustion among nurses. The aim was to improve emotional regulation and decrease sympathetic nervous system activation.	44 nurses 90.9% female aged 27–54 years	Nurses from the hospital center	RCT	IG = 22 CG= 22	“white ball” Qigong system (Biofeedback). Training: 2 supervised sessions weekly (5 minutes each) for 4 weeks, followed by self-practice twice daily for another 4 weeks. Participants had to practice 5 static postures focusing on breathing, relaxation, and visualization, aiming to reduce stress. CG: has not received any intervention.	**Mental Health and Psychological**: Emotional exhaustion (EE), depersonalization, perception of personal accomplishment: MBI	The Qigong group showed a significant reduction in emotional exhaustion (EE) scores compared with the control group. The dimensions of depersonalization and personal accomplishment did not show significant changes (*P* >.05).
Lemaire (32); Canada; 2011	Determine whether the use of a biofeedback-based stress management tool combining rhythmic breathing, self-generated positive emotions, and feedback helps to reduce physician stress.	40 (23 men and 17 women) age 44.8 ± 8.2 (control) and 47.8 ± 8.5 (intervention)	staff physicians (1 from primary care, 30 from a medical specialty and 9 from a surgical specialty) from urban tertiary care centers	RCT	IG = 21 CG= 19	(Rhythmic breathing with positive emotions) Biofeedback. Training: to use the stress management tool during study days 28 to 56 for 5 minutes at least three times daily. The participants were offered the opportunity to request and receive additional training and support. Control group did not receive reinforcement in the use of stress	**Mental Health and Psychological** Stress: PSS (15 items) Coherence, Energy, Fatigue, irritability, Depression, Mental Clarity (or Concentration), Sleep Quality, Perceived Stress at Work, Communication: POQA-R **physiological** HR and BP: Physiologic Auto Blood Pressure Monitor model 106-925 Salivary Cortisol: Salivary Cortisol Analysis (Salimetrics LLC, State College) enzyme immunoassay	PSS: Significant post-intervention reduction in the experimental group (p <.05). Other Mental Health and Psychological outcomes: Significant improvements (p <.05) in energy, irritability, depression, sleep quality, and communication (intervention group only). Physiological outcomes: Significant reduction in cortisol levels in the intervention group, indicating lower physiological stress.
Macedo (33); Brazil; 2023	Evaluate whether cardiovascular biofeedback (CBKF) training could increase HRV, improve autonomic nervous system regulation, and reduce stress in nurses.	115 83.5% female Age mean 43.2 years (SD± 8.4)	Nursing professionals (nurse; technician; assistant) with stress symptoms, from a university hospital	RCT	IG = 58 CG= 57	Cardiovascular biofeedback (CBKF) training Training: 9 sessions, 10 minutes, 3 times a week, over 3 weeks, using EmWave Pro Plus^®^ software for real-time HRV monitoring and training. The participant slow-paced breathing at six breaths per minute, guided by interactive software games. Control group performed online puzzles called Jigsaw on the tablet, without self-monitoring.	**Mental Health and Psychological**: Overall Stress Level (OSL): SSS Occupational stress: WRSS **Physiological**: HRV, including: SDNN (standard deviation of NN intervals); rMSSD (root mean square of successive differences); LF/HF ratio (Low/High Frequency); Cardiac coherence: EmWave Pro Plus^®^ (with earlobe photoplethysmograph)	Significant increases in SDNN (p = 0.016) and LF/HF ratio (p < 0.001) were observed in the intervention group, reflecting improved autonomic regulation and stress adaptability following cardiovascular biofeedback training.
Hsieh (27); Taiwan; 2020	Compare the effectiveness of Biofeedback training BT and a SDBT (SDBT) on work stress, depressive symptoms, resilience, heart rate variability HRV, and respiration rate among psychiatric nurses who experienced workplace violence.	135 119 females (88.1%) and 16 males (11.9%) Age mean 35.61 years (SD = 8.16)	Psychiatric nurses exposed to workplace violence from three hospitals	Quasi-experimental study with randomized cluster sampling	IG = 49 BT IG=47 SDBT CG= 39	Biofeedback Training: weekly 60-minute sessions for 6 weeks. The participant’s focus was on muscle relaxation, diaphragmatic breathing and real-time respiratory sinus arrhythmia (RSA) biofeedback. SDBT Training: once a week for six weeks. Participants were provided with an MP4 video containing guided short meditation practices and real-time biofeedback. The control group did not receive the BT or SDBT intervention.	**Mental Health and Psychological**: Depressive symptoms: CES-D Resilience: RS Work stress: OSI-2 **Physiological**: standard deviation of normal to normal (SDNN), LF, HF, and HRV: ECG.	Mental Health and Psychological outcomes: Both experimental groups showed significant improvements in depressive symptoms, resilience, and respiratory rate compared to the control group (p <.05). Occupational stress: The Smartphone-Delivered Biofeedback Training (SDBT) group reported a significant reduction in work-related stress (p = 0.013) compared to both the control group (p = 0.723) and the traditional Biofeedback Training (BT) group (p = 0.072).
Orlando (31); USA; 2001	To examine the effectiveness of a self-regulation training program with Biofeedback in reducing perceived stress and improving job satisfaction among hospital-based primary care professionals	18 Age Mean GI = 44.0 years (SD 7.7) GC = 40.0 (SD 9.8)	Physicians, nurse practitioners, and nurses from two family medicine clinics	Pilot study quasi−experimental study	IG = 9 CG= 9	(Self-Regulation) Biofeedback The training consisted of 5 minutes of daily self-regulation using the emWave Pro device for 12 weeks, with weekly peer support sessions provided during the first 6 weeks. All participants received a 1-hour introductory session on the rapid coherence technique and practiced it. The control group received the same training without peer support.	**Mental Health and Psychological**: Stress: PSS Job satisfaction: MSQ−SF **Physiological**: Cardiac coherence (weekly logs, not formally measured).	A significant initial increase in perceived stress was observed in both groups, more pronounced in the treatment group (p = 0.03). Participants in both groups reported independent use of self-regulation techniques, although many described difficulties related to time constraints, feelings of overload, and challenges in maintaining regular practice.
Balk (41); USA; 2009	To evaluate whether relaxation training reduces stress levels and has a physiological and Mental Health and Psychological impact	90 46 OR mean age 42 ± 9.5); 14 non-OR mean age 38 ± 8.7	- operating room (OR) nurses and staff (mean age 42 ± 9.5); - non-OR hospital employees (mean age 38 ± 8.7); - 30 oncology patients (mean age 50 ± 10.2) In the University hospital	Pre-post intervention study with group comparisons (non-randomized, no control group).	GI: OR staff (*n* = 46) Non-OR employees (*n* = 14) Cancer patients (*n* = 30) No control groups	Biofeedback 30-minute relaxation training. The biofeedback technique used Biodot as a tool. Participants were involved in meditation and relaxation sessions, guided imagery, written dialogue and genograms.	**Mental Health and Psychological**: Stress: PSS **Physiological**: Biodot: adhesive that changes color with skin temperature, indicating relaxation via biofeedback	All groups showed significant reductions in perceived stress (PSS) and increases in skin temperature, indicating decreased sympathetic activity (p <.05). The operating room (OR) staff, who initially exhibited higher stress levels, demonstrated significant post-intervention improvements, confirming the effectiveness of the training as a brief intervention for both subjective and physiological stress.
Allen (42); Australia; 2020	To measure the impact of meditation with neurofeedback on brainwave regulation, physiological stress (cortisol), and subjective well-being in O&G doctors.	12 female (75%) age 25–35: median training time 4.5 years	obstetrics and gynaecology (O&G) doctors and labour ward teams in metropolitan hospitals.	Mixed-methods exploratory study, pre-post design without a control group.	GI: 12 No control group	Neurofeedback The training consisted of assisted meditation using the Muse mobile application EEG headband, 20 minutes daily for 21 consecutive days. Real-time audio feedback guided participants in achieving meditative brainwave patterns.	**Mental Health and Psychological**: Depression, Anxiety and Stress: DASS-21; perceived situational Stress: VSS; subjective well-being and emotional regulation: qualitative interviews. **Physiological**: physiological stress response: Salivary cortisol; brainwave regulation: Muse EEG (alpha wave activity)	Mental Health and Psychological outcomes: Meditation combined with neurofeedback produced significant reductions in situational stress and anxiety, as indicated by lower DASS-21 scores (p <.05). Participants also reported enhanced focus, emotional regulation, and relaxation during clinical tasks.
Cutshall (34); USA; 2011	To evaluate the effectiveness of a self-directed, computer-guided meditation training program using biofeedback in reducing stress among hospital nurses.	11 (8 completed the Intervention) aged 23–61 years (median age = 44)	nurses in Mayo Clinic hospital	Pilot prospective pre-post study without a control group	GI= 8 No control group	Biofeedback The Training consisted of assisted meditation using the Healing Rhythms program (Wild Divine software), 30 minutes to session, 4 times/week for 4 weeks at home. Participants had to follow 15 structured and guided modules, as well as optional breathing and relaxation exercises.	**Mental Health and Psychological**: Perceived vitality/fatigue: SF-36; State and trait anxiety: STAI; self-rated: LASA.	The intervention group showed significant reductions in anxiety (p <.03) and stress levels (p <.01), along with improvements in vitality (p <.04). Participants, primarily nurses, reported high satisfaction with the program (mean score = 8.6/10). The intervention was feasible, well-tolerated, and effective in reducing Mental Health and Psychological stress among hospital nurses.
Mensinger (29); USA; 2024	To examine the feasibility, acceptability, and preliminary efficacy of a rate variability biofeedback (HRVB) smartphone app for improving well-being in HCWs during the COVID-19 pandemic.	28 89% female mean age = 45.6 years; range 28–65	Nurses (79%), Physicians, EMTs (and Chaplains) in to Healthcare institutions	Pilot single-arm, non-randomized feasibility study with pre-, mid-, and post-assessment.	GI= 28 No control group	Heart Rate Variability Biofeedback (HRV-B) smartphone app Training: 10–20 minutes of daily practice for 6–8 weeks, with weekly virtual check-ins. Participants used the app to breathe at their individual resonance frequency and increase HRV.	**Mental Health and Psychological**: - Perceived stress: PSS; -psychological resilience/sense of coherence: SOC-R; -mindful self-care behaviors: MSCS-B; -body appreciation: BAS-2; -interoceptive awareness: MAIA; -loss of control over eating: LOCES -eating disorder symptoms: EDE-Q7. **Physiological**: parasympathetic activity/autonomic nervous system regulation: HRV (RMSSD (Root Mean Square of Successive Differences)).	Participants reported that HRV biofeedback (HRVB) promoted relaxation and improved stress management, as well as greater body awareness, intuitive eating, self-care, and resilience. Physiological findings showed a significant increase in HRV (RMSSD), indicating enhanced autonomic regulation. The intervention was feasible, well-accepted, and associated with high engagement.
Ribeiro (30); Spain; 2023	Assess the effectiveness of HRV biofeedback (HRV-BF) protocol in mitigating mental health symptoms in a sample of frontline HCWs (HCWs) during COVID-19 pandemic.	21 Female aged 19–59 years (mean age 37.7 ± 11.7 years)	Clinicians 6 (28.6%) Nurses 13 (61.9%) Auxiliary nurses 2 (9.5%) from two tertiary hospitals.	Single-group pre-post experimental study using two psychological assessment approaches.	GI= 21 No control group	Heart Rate Variability Biofeedback (HRV-BF) The training consisted of resonance-frequency breathing, heart-rhythm coherence exercises, and real-time HRV feedback using the NeXus-10 MKII system. Participants attended five weekly 45–60 min in-person sessions and engaged in daily home practice supported by instructional materials.	**Mental Health and Psychological**: - subjective stress intensity: VASS; - perceived stress: PSS - PTSD symptoms: PCL-5; - state anxiety: STAI-S - trait anxiety:STAI-T - depressive symptoms: PHQ9 **Physiological**: Autonomic nervous system regulation: HRV (SDNN, LF power) via NeXus-10 MKII Breathing control/parasympathetic activation: respiratory rate via NeXus-10 MKII; Sympathetic arousal: phasic EDA via NeXus-10 MKII	HRV-BF training significantly reduced chronic stress, anxiety, and PTSD-related symptoms, accompanied by increased HRV (SDNN, LF power) and decreased respiratory rate, consistent with parasympathetic activation. Participants showed high engagement and adherence. Phasic EDA indicated transient sympathetic arousal during sessions, suggesting adaptive autonomic recalibration.
Park (43); USA; 2018	To examine the effects of a wearable biofeedback postural training device on pain and posture in workers with pre-existing low back pain caused by prolonged static posture (e.g., sitting).	31 13 men, 18 women; age = 33.1 _ 13.3 years old	Hospital administrative staff and laboratory workers with chronic low back pain due to static posture.	Randomized Controlled Trial (RCT)	IG= 15 CG= 16	Wearable posture biofeedback Training: Postural correction through vibratory biofeedback delivered in real time via a wearable device placed on the lumbar spine, using Upright Go/LumoBack. Participant Tasks: Wear the device for 1 hour per day, 5 days per week, over 4 weeks to improve posture during daily activities. Control group received no postural feedback.	**Mental Health and Psychological**: Pain intensity: VAS **Physiological/postural:** Postural alignment and spinal angles: 3D photogrammetry Posture metrics (HFA, shoulder angle, trunk angle): postural angle measurements	Wearable postural biofeedback significantly reduced low back pain (VAS; p <.05) and improved postural alignment, as indicated by decreased trunk and head angles. The intervention was well-tolerated and accepted, confirming its effectiveness in enhancing musculoskeletal well-being among sedentary HCWs.

Tools: State-Trait Anxiety Inventory, Y form (STAI-Y), S form (STAI-S), T form (STAI-T); 36-Item Short Form Health Survey (SF-36) Maslach Burnout Inventory (22 ITEM) (MBI); Personal and Organizational Quality Assessment–Revised (POQA-R) questionnaire (25 items); Work-Related Stress Scale (WRSS); Centre for Epidemiological Studies Depression (CES-D) Chinese version; Resilience Scale (RS) Chinese version; Occupational Stress Indicator (OSI-2); Minnesota Satisfaction Questionnaire Short Form (MSQ−SF); Visual Stress Scale (VSS); Linear Analogue Self-Assessment (LASA); Sense of Coherence Scale–Revised (SOC-R; Mindful Self-Care Scale–Brief (MSCS-B); Body Appreciation Scale-2 (BAS-2); Multidimensional Assessment of Interoceptive Awareness (MAIA); Loss of Control Over Eating Scale–Brief (LOCES); Eating Disorder Examination Questionnaire–Short (EDE-Q7); Visual Analog Stress Scale (VASS); Post-traumatic Stress Disorder Checklist for DSM-5 (PCL-5); Patient Health Questionnaire-9 (PHQ9); Visual Analogue Scale (VAS). Bold values indicate statistically significant between-group or pre–post differences (p < 0.05).

### Search strategy

The search strategy included the following keywords: Biofeedback, Self-regulation, Well-being, Work-related stress, Healthcare professionals, job stress, burnout, occupational health, workload, job satisfaction, occupational. These were combined to construct specific search strings. The search strategy combined controlled vocabulary terms and free-text keywords related to biofeedback, occupational stress, and healthcare professionals using Boolean operators (AND/OR). Search strings were adapted to the specific syntax and indexing systems of each database (e.g., MeSH terms for PubMed, Emtree terms for Embase), while maintaining conceptual consistency across sources. A complete example of the search strategy is provided in the **Supplementary Material**. In the supplementary document, we report an example of a complete string used in PubMed, one of the main search engines (as shown in **Supplementary Table S1**).

### Study selection

Two reviewers (EC and AU) independently screened titles and abstracts using a standardized Excel form. Duplicate records were removed before screening. Potentially eligible studies were retrieved in full text and assessed independently by the same reviewers.

Discrepancies were resolved through discussion; when consensus was not reached, a third reviewer (GC) adjudicated.

The selection procedure is detailed in the PRISMA 2020 flow diagram (**Supplementary Figure S1).**

### Data collection process

Data extraction was performed independently by two reviewers (EC and AU) using a piloted and standardized extraction sheet. Extracted data included: study characteristics (authors, year, country, study design, aim); population characteristics (type of healthcare workers, age, sample size); intervention characteristics (e.g., EMG-BF, HRV-BF, neurofeedback; duration, frequency, comparators); outcome domains, including: mental health and psychological outcomes (stress, burnout, anxiety, depression, psychosomatic symptoms, pain, job satisfaction, resilience, well-being, work efficiency); physiological outcomes (HRV parameters, heart rate, blood pressure, skin conductance, salivary cortisol); measurement instruments; main results (direction and magnitude of effects, p-values, effect sizes).

Any discrepancies in the extracted data were discussed among reviewers and, when needed, verified by the supervisor (GC). Authors were not contacted for missing data, and no automation tools were used for the extraction process. Information on funding sources or conflicts of interest of the primary studies was not collected. Assumptions were minimized and made only when judged methodologically reasonable based on the available information.

The complete list of extracted data items is reported in **Supplementary Table S1**.

### Risk of bias assessment

The risk of bias was evaluated at the study level using established and widely accepted methodological guidance for assessing internal validity, which were chosen beforehand based on the study design (44). Randomized controlled trials (RCTs) (32, 33, 39, 40, 43) were evaluated using a domain-based risk of bias approach, whereas non-randomized and pre-post studies (27, 29–31, 34, 41, 42) were evaluated using the ROBINS-I tool (45). This tool examines biases from confounding factors, participant selection, intervention classification, deviations from planned interventions, missing data, outcome measurement, and selective reporting.

For RCTs, risk-of-bias assessments across domains were visually summarized with the robvis package in R (version 3.0), used solely for graphical display (see **Supplementary Materials**). The overall risk of bias was classified as low, moderate (some concerns), or high, depending on the number and severity of domains rated as at risk.

Of the 12 included studies, 5 RCTs (32, 33, 39, 40, 43) exhibited a moderate risk of bias. Conversely, all non-randomized and pre–post studies (27, 29–31, 34, 41, 42) raised significant methodological issues, mainly due to uncontrolled confounding factors, reliance on self-reported outcomes, and inadequate handling of missing data.

### Summary measures

Principal summary measures included mean differences, percentage improvements, p-values, standardized effect sizes (e.g., Cohen’s d), and physiological indicators such as HRV and salivary cortisol.

### Synthesis of results

A synthesis table (**Supplementary Table S2**) was created that includes study characteristics, interventions, outcomes, and main results. Due to heterogeneity in design, outcome measures, and reported data, a meta-analysis was not feasible. Therefore, results were synthesized qualitatively.

Several methodological weaknesses across the included studies warrant explicit consideration, including small sample sizes, uncontrolled pre–post designs, and the frequent absence of active comparators. In addition, some studies reported null or discordant findings, particularly with respect to subjective stress measures or selected physiological outcomes, underscoring the inconsistency of effects across domains.

Comparators varied substantially, ranging from no intervention to stress diaries, relaxation exercises, or alternative behavioral activities, further contributing to heterogeneity and limiting cross-study comparability.

This methodological diversity posed significant challenges for synthesis, requiring a structured narrative approach that prioritized critical appraisal and contextual interpretation over formal aggregation. The synthesis process therefore involved balancing heterogeneous designs, outcomes, and risk-of-bias profiles to provide an accurate and cautious representation of the current evidence base.

Patterns of consistency and variation among interventions and outcomes were described narratively. No subgroup or sensitivity analyses were conducted, and no missing data were imputed.

## Results

### Descriptions of studies

Initially, 3,564 records were identified via electronic database searches, and 5 more via citation searches. After removing 207 duplicates and excluding 3,328 non-matching records during screening, four full texts couldn’t be retrieved, and three more articles failed to meet the criteria. In total, 12 studies were included (**Supplementary Figure S1**).

The analysis of the studies showed that the Biofeedback interventions examined were diverse: HRV-BF (29, 30); RSA-BF (39); CBKF (33); EEG-BF (42); Biofeedback using Smartphone (SDBT) (27); Biofeedback through the Healing Rhythms meditation program (34); Wearable posture Biofeedback (43); and finally, Biofeedback combined with other techniques (30–32, 40, 41). All studies aimed to improve HCWs health and reduce stress symptoms through structured programs. Five studies (32, 33, 39, 40, 44) conducted randomized controlled trials (RCTs), comparing an experimental group with a control group; two (27, 31) were quasi-experimental studies, one of which was a pilot study; and five (29, 30, 34, 41, 42) were pre-post intervention studies with or without a control group. All studies aimed to improve HCWs’ health and reduce stress symptoms through structured programs. They mainly involved workers experiencing stress-related symptoms. Methodological quality was mostly moderate to low, due to small sample sizes and lack of blinding.

### Mental health and psychological measures

*Stress, Job Satisfaction, and Burnout:* Eight studies (29–34, 41, 42) used various tools to assess perceived stress, including the Perceived Stress Scale (PSS) (29–32, 41), the Stress Symptoms Scale (SSS) (33), the Depression Anxiety Stress Scale (DASS-21) (42), the Visual Stress Scale (VSS) (42), and the Linear Analogue Self-Assessment (LASA) (34).

Across these studies, significant reductions in stress were consistently observed following biofeedback interventions (p <.05) (30, 32, 34, 41, 42). One study (33) reported no change in self-perceived stress but a clear physiological improvement in HRV. The other (27) found that smartphone-delivered biofeedback (SDBT) produced stronger reductions in occupational stress (p = .013) than both traditional biofeedback and the control condition. One study (40) observed that Qigong combined with biofeedback significantly reduced emotional exhaustion among nurses, though other burnout dimensions remained unchanged. Job satisfaction was examined in a few studies: one (31) noted near-significant post-intervention improvements (p = .06–.07).

*Anxiety, Depression, and Well-Being:* The studies evaluated anxiety using the State-Trait Anxiety Inventory (STAI-Y) (34, 39) or the DASS-21 (42). Significant decreases were observed in two studies (34, 39) for trait anxiety (p = .004) and for state and trait anxiety (p = .03). One author (42) reported lower anxiety and stress scores following neurofeedback training, indicating improved mood and emotional regulation.

Depression was assessed with the Centre for Epidemiological Studies Depression (CES-D), the PHQ-9, or the DASS-21 in three studies (27, 30, 42), all of which reported significant post-intervention reductions.

Combined programs, biofeedback plus breathing, mindfulness, or relaxation, showed the strongest and most consistent psychological improvements (27, 30, 34).

Two studies using the 36-Item Short Form Health Survey (SF-36) (34, 39) reported increases in vitality, social functioning, and perceived health. At the same time, another author (29) found enhanced resilience, interoceptive awareness, and self-care using an HRV biofeedback smartphone app.

Collectively, these results highlight biofeedback’s role in reducing stress, anxiety, and depressive symptoms while fostering emotional balance and well-being.

*Pain and Physical Functioning:* The RCT (43) demonstrated that wearable postural biofeedback significantly reduced low-back pain and improved spinal alignment (p <.01), suggesting promising applications for musculoskeletal and occupational health in sedentary healthcare staff.

### Physiological measures

The HRV emerged as the primary indicator of parasympathetic regulation. Several studies reported a significant increase in HRV after biofeedback training (29, 30, 33, 39), signaling improved autonomic balance and cardiorespiratory coherence. These results align with enhanced self-regulation and relaxation abilities, often achieved through slow, diaphragmatic breathing techniques (27, 30, 33, 39). Findings regarding HR, BP, and salivary cortisol were less consistent: multiple studies showed no significant changes (33, 39, 42), although one reported a significant decrease in cortisol levels in the intervention group (32). Similarly, EEG alpha activity showed no notable differences (42). Conversely, some studies found a significant decrease in skin conductance level (SCL) and an increase in skin temperature, indicating reduced sympathetic arousal (39, 41). Discrepancies appeared between physiological and subjective measures: for example, certain studies reported improvements in HRV without corresponding reductions in perceived stress (33), while others noted psychological benefits without changes in biomarkers (42). Lastly, one study demonstrated that wearable postural biofeedback notably improved posture and alleviated low back pain (43), highlighting the potential of this technology for musculoskeletal and postural modulation.

In summary, the physiological findings reveal a consistent pattern across studies: increased HRV, decreased SCL and respiratory rate, and improved autonomic balance, all of which support the psychological benefits associated with biofeedback interventions.

### Quality assessment

The RCTs showed a moderate-to-low risk of bias, with appropriate randomization procedures and outcome assessments supporting the reliability of their findings (32, 33, 39, 40, 43). In contrast, pre-post and non-randomized studies were at serious risk of bias, primarily due to uncontrolled confounding, reliance on self-reported measures, and inadequate handling of missing data (27, 29–31, 34, 41, 42). The absence of control groups and formal randomization further limits causal inferences, while the use of subjective outcomes without blinding may have increased response bias. Additionally, incomplete reporting on attrition and data management raises concerns about the generalizability of results. Despite these methodological weaknesses, most studies provided clear descriptions of interventions, reported outcomes transparently, and offered preliminary but promising evidence that biofeedback can enhance well-being and stress regulation among HCWs.

## Discussion

This systematic review shows that biofeedback interventions, especially HRV-BF and RSA-BF, have potential in supporting healthcare professionals’ psychophysiological well-being. In the included studies, these interventions were linked to improved parasympathetic regulation and reductions in perceived stress, anxiety, and depressive symptoms, helping achieve greater emotional balance (31–33, 39, 41–43).Although effect sizes and outcome measures varied, the direction of findings was consistent with previous evidence showing that biofeedback may help mitigate chronic stress and enhance resilience across both clinical and non-clinical populations.

Furthermore, interventions that integrated biofeedback with slow-breathing, mindfulness, or relaxation techniques tended to produce more stable and lasting benefits than standalone biofeedback protocols (27, 30, 33, 39), as reported by the respective study authors.

The observed improvements can be interpreted within contemporary psychophysiological models, in which enhanced HRV and respiratory synchrony promote greater vagal activation, reduced sympathetic arousal, and improved emotional self-regulation.

Taken together, these converging psychological and physiological effects suggest that biofeedback may represent a promising avenue for strengthening resilience and mitigating the burden of occupational stress in healthcare environments.

### Quality and limitations of evidence

Although the results are promising, the quality of the available evidence remains limited. Only five out of twelve studies were randomized controlled trials (RCTs), while the remainder employed pre–post or quasi-experimental designs without control groups. Sample sizes were generally small and mainly composed of nursing professionals, limiting representativeness. Mental Health and Psychological outcomes were measured with heterogeneous tools, such as the PSS, the DASS-21, and the VSS, which reduces comparability across studies. Importantly, the majority of included studies were characterized by small sample sizes and uncontrolled pre-post designs, which substantially limit internal validity and preclude robust causal inference. While the direction of findings appears broadly consistent, the current evidence should be interpreted as preliminary and hypothesis-generating rather than confirmatory. Accordingly, biofeedback interventions cannot yet be considered as having established efficacy in this population, underscoring the need for adequately powered randomized controlled trials. Furthermore, some studies lacked follow-up evaluations or objective physiological measures, while others relied solely on self-reported outcomes (29, 42, 43), increasing the risk of bias. A further limitation of this review is the absence of a formal assessment of the certainty of evidence, such as the GRADE approach. Given the substantial heterogeneity in study designs, interventions, and outcome measures, applying a standardized certainty framework was not methodologically appropriate. Consequently, conclusions should be interpreted as indicative rather than definitive, reinforcing the need for larger, well-designed randomized trials with standardized outcomes. The generalizability of the present findings is further constrained by the predominance of nursing populations and the limited representation of other healthcare professionals, such as physicians, allied health workers, and administrative staff. Moreover, the scarcity of multicenter studies restricts the external validity of the evidence across diverse healthcare systems and organizational contexts. It should be acknowledged that the limited number of methodologically acceptable studies and their pronounced heterogeneity required a narrative systematic review approach rather than a fully standardized systematic synthesis. Although this methodological framing is not explicitly reflected in the title, it was deliberately adopted to ensure coherence with the scope and structure of the available evidence. This choice prioritizes transparency and critical appraisal over formal aggregation, allowing a balanced interpretation of findings while avoiding overstatement of evidentiary strength. Future research should prioritize multicenter designs and more heterogeneous professional samples to enhance the applicability of biofeedback interventions in occupational health settings.

### Strengths and methodological considerations

Although a meta-analysis was not possible due to data heterogeneity and small sample sizes, the narrative synthesis provided an integrated understanding of both Mental Health and Psychological and physiological outcomes. A major strength of this review is that it represents the first systematic synthesis specifically focused on the application of biofeedback interventions among healthcare professionals, offering a comprehensive overview of both psychological and physiological outcomes.

A key strength is the inclusion of various biofeedback modalities, offering a comprehensive overview of emerging applications, from HRV and respiration-based protocols to neurofeedback and wearable devices. However, the lack of long-term follow-up data limits the ability to determine whether participants maintained their self-regulation skills over time.

### Implications for clinical practice and occupational health

The interpretation of the findings is limited by substantial heterogeneity across study designs, biofeedback modalities, outcome measures, small sample sizes, and the predominance of nursing staff, which restricts the generalizability of the results. Despite these limitations, the evidence suggests that biofeedback is a valuable, non-invasive, and feasible strategy for managing work-related stress and promoting resilience among HCWs. Its integration into psychoeducational and training programs could reduce burnout, improve job satisfaction, and enhance the quality of patient care. The emergence of portable and smartphone-based biofeedback systems expands accessibility and practicality in dynamic healthcare environments, where traditional psychological interventions are challenging to implement. Such interventions could be incorporated into occupational health and psychosocial risk prevention programs, in line with European directives and the Italian Legislative Decree 81/2008 on workplace safety. Future research should adopt a more standardized, integrative approach, combining Mental Health and Psychological, physiological, and occupational measures to capture the multidimensional impact of biofeedback fully. Large, multicenter randomized controlled trials are needed to verify the stability of results over time and to compare the effectiveness of different biofeedback modalities, such as HRV-BF, RSA-BF, neurofeedback, and postural feedback.

Although the methods and results of this review are coherent and internally consistent, the overall level of evidence supporting biofeedback interventions in healthcare professionals remains limited. The predominance of small, heterogeneous, and non-controlled studies necessitates a conservative interpretation, in which observed benefits should be viewed as indicative signals rather than definitive evidence of effectiveness. Accordingly, conclusions are framed to emphasize uncertainty and the need for more rigorous, adequately powered trials.

An important practical consideration emerging from this review concerns the context in which biofeedback interventions were delivered. Most included studies implemented training protocols outside regular working hours or in controlled settings, often as scheduled sessions or self-directed home practice. Only a limited number of interventions approximated real-life workplace application, such as brief sessions integrated into the workday or the use of portable or wearable biofeedback devices; this distinction is relevant for interpreting feasibility and ecological validity, as interventions delivered outside working hours may introduce selection bias and limit scalability. Future research should prioritize the evaluation of biofeedback methods embedded within routine clinical workflows to better reflect real-world occupational settings.

Equally important is developing transparent, replicable intervention frameworks, ensuring that methodologies, training protocols, and adherence-monitoring procedures are clearly documented. This will enable future studies to refine biofeedback applications and establish evidence-based programs suitable for clinical and occupational health settings. Such improvements are crucial to establishing a gold-standard biofeedback protocol for healthcare professionals, providing evidence-based strategies to prevent burnout and improve overall well-being in clinical and occupational environments.

## Conclusion

Evaluating the effectiveness of Biofeedback interventions is essential for developing standardized programs to manage work-related stress and burnout, ultimately enhancing organizational climate and HCWs’ confidence in managing their own health. A structured intervention framework is required to promote positive perceptions of care, support self-satisfaction in the work environment, and enable individuals to function as competent and efficient professionals.

Despite encouraging findings, the evidence remains preliminary due to methodological heterogeneity and the lack of standardized protocols or long-term follow-up. Large, multicenter randomized controlled trials are urgently needed to establish robust, reproducible intervention models for implementation in occupational health settings.

## Data Availability

The original contributions presented in the study are included in the article/**Supplementary Material**. Further inquiries can be directed to the corresponding author.
