# Supplementary material for: Enhancing wellness: a systematic review of biofeedback interventions for healthcare professionals
Source: Front Psychiatry. 2026 Mar 16;17:1761371. doi: 10.3389/fpsyt.2026.1761371 (PMC13033714; doi:10.3389/fpsyt.2026.1761371)
Supplement: Supplementary file 1 [file Supplementaryfile1.docx]

**Supplementary Materials**

This section reports the table cited in the review’s full text.

| PubMed | *(work related stress[Title/Abstract]) AND (Biofeedback [Title/Abstract]); (Occupational Stress[Title/Abstract]) AND (Biofeedback [Title/Abstract]; (Job Stress [Title/Abstract]) AND (Biofeedback [Title/Abstract])); (Burnout [Title/Abstract]) AND (Biofeedback [Title/Abstract]); (Occupational Health [Title/Abstract]) AND (Biofeedback [Title/Abstract]); (Occupational Health [Title/Abstract]) AND (Biofeedback [Title/Abstract]); (Workload [Title/Abstract]) AND (Biofeedback [Title/Abstract]); (Job Satisfaction[Title/Abstract]) AND (Biofeedback [Title/Abstract]); (Occupational [Title/Abstract]) AND (Biofeedback [Title/Abstract]); (organizational [Title/Abstract]) AND (Biofeedback [Title/Abstract]); (workplace [Title/Abstract]) AND (Biofeedback [Title/Abstract]); (hospital employ* [Title/Abstract]) AND (Biofeedback [Title/Abstract]); (Hospital [Title/Abstract]) AND (Biofeedback [Title/Abstract]); (worker*[Title/Abstract]) AND (Biofeedback [Title/Abstract]); (healthcare worker*[Title/Abstract]) AND (Biofeedback [Title/Abstract]); (healthcare worker [MeSH Terms]) AND (Biofeedback [Title/Abstract]).* |
| --- | --- |

Table S1. Strings PubMed

|  |
| --- |
|  |

Figure S1 PRISMA 2020 flow diagram for new systematic reviews, which included searches of databases, registers, and other sources


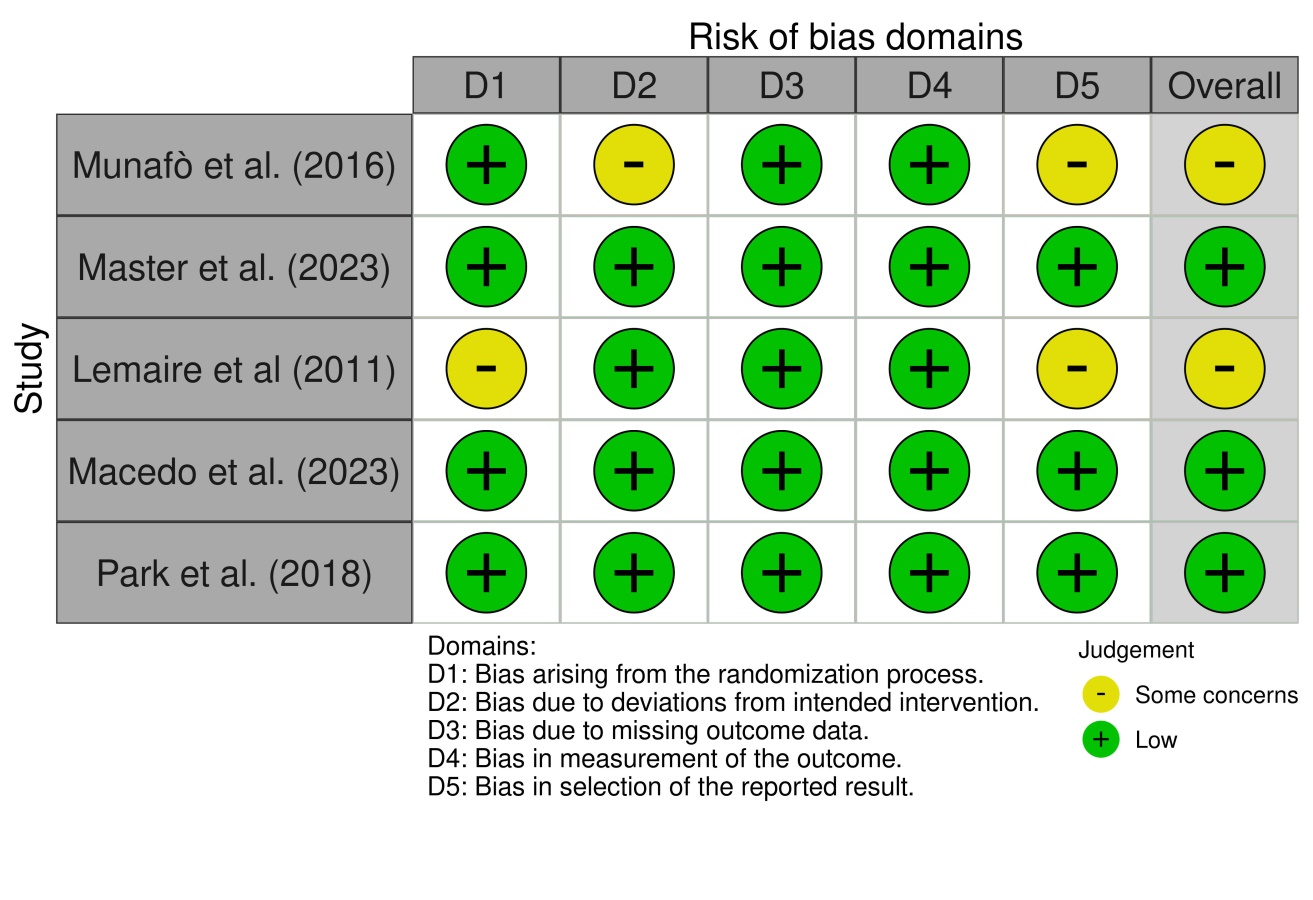


Figure S2. Traffic light plot RCTs.

| Study | D1 Confounding | D2 Selection | D3 Classification | D4 Deviation | D5 Missing data | D6 Measurement | D7 Reported result | Overall |
| --- | --- | --- | --- | --- | --- | --- | --- | --- |
| Hsieh | Serious | Serious | Low | Moderate | Serious | Serious | Low | Serious |
| Mensinger | Serious | Serious | Low | Moderate | Serious | Serious | Low | Serious |
| Castro Ribeiro | Serious | Moderate | Low | Moderate | Serious | Serious | Low | Serious |
| Allen | Serious | Serious | Low | Moderate | Serious | Serious | Low | Serious |
| Cutshall | Serious | Serious | Low | Moderate | Serious | Serious | Low | Serious |
| Balk | Serious | Serious | Low | Low | Serious | Serious | Low | Serious |
| Orlando | Serious | Moderate | Low | Moderate | Serious | Serious | Low | Serious |

Figure S3. Graphical summary of observational and non-randomized studies.
